# Clarifying ethical stances in conservation: a trolley problem thought experiment

**DOI:** 10.1093/biosci/biaf052

**Published:** 2025-06-17

**Authors:** Guillaume Latombe, Ugo Arbieu, Sven Bacher, Stefano Canessa, Franck Courchamp, Stefan Dullinger, Franz Essl, Michael Glaser, Ivan Jarić, Bernd Lenzner, Anna Schertler, John R U Wilson

**Affiliations:** Institute of Ecology and Evolution, The University of Edinburgh, Edinburgh, Scotland, United Kingdom; Université Paris-Saclay, CNRS, AgroParisTech, Ecologie Société Evolution, Gif-sur-Yvette, France; Department of Biology, University of Fribourg, Fribourg, Switzerland; Division of Conservation Biology, Institute of Ecology and Evolution, University of Bern, Bern, Switzerland; Dipartimento di Scienze e Politiche Ambientali, Universitá degli Studi di Milano, Milan, Italy; Université Paris-Saclay, CNRS, AgroParisTech, Ecologie Société Evolution, Gif-sur-Yvette, France; Division of Biodiversity Dynamics and Conservation, Department of Botany and Biodiversity Research, University of Vienna, Vienna, Austria; Division of BioInvasions, Global Change, and Macroecology, Department of Botany and Biodiversity Research, University of Vienna, Vienna, Austria; Division of BioInvasions, Global Change, and Macroecology, Department of Botany and Biodiversity Research, University of Vienna, Vienna, Austria; Université Paris-Saclay, CNRS, AgroParisTech, Ecologie Société Evolution, Gif-sur-Yvette, France; Biology Centre of the Czech Academy of Sciences, Institute of Hydrobiology, České Budějovice, Czech Republic; Division of BioInvasions, Global Change, and Macroecology, Department of Botany and Biodiversity Research, University of Vienna, Vienna, Austria; Division of BioInvasions, Global Change, and Macroecology, Department of Botany and Biodiversity Research, University of Vienna, Vienna, Austria; Vienna Doctoral School of Ecology and Evolution, University of Vienna, Vienna, Austria; South African National Biodiversity Institute, Kirstenbosch Research Centre, Cape Town; Centre for Invasion Biology, Department of Botany and Zoology, Stellenbosch University, Stellenbosch, South Africa

**Keywords:** conservation, ethics, management, metaphor, trolley problem

## Abstract

Conservation policies often need to integrate scientific predictions with ethical considerations. However, different normative ethical systems at the root of conservation approaches often support different decisions, and the moral stances of stakeholders are influenced by diverse societal values and perceptions. This creates the potential for dilemmas and conflicts. In the present article, we adapt the well-known trolley problem thought experiment to a conservation context. Exploring variations in how the problem is framed enables us to highlight key concepts that need to be considered in decision-making (uncertainty; asymmetry in numbers, victims, and impacts; temporal and spatial asymmetry; causal relationships and stakeholder involvement). We argue that the trolley problem offers a simplified but flexible framework to understand and predict the factors underlying differences in moral stances across diverse conservation issues, foster communication, and facilitate informed decision-making in conservation practice.

Conservation practitioners often have to make decisions about actions that are believed to benefit one entity of nature (*sensu* Johnson 1983 and Lehnen et al. 2022; e.g., a group of individuals, populations, species, or ecosystems; see box 1 for definitions) but may be detrimental for another. These decisions are rarely straightforward. Consequences are often uncertain, and trade-offs can be perceived differently by stakeholders, because of ecological, economic, political, cultural, philosophical, or psychological factors (Bennett 2016). Conservation decisions must therefore account for the diversity of nature's values and relationships between people and the environment (Ives and Kendal 2014). In particular, modern conservation approaches advocate for a more consistent consideration of local cultures to engage local actors, forge new partnerships, and make conservation relevant to people (Infield and Mugisha 2013). Consequently, conservation must consider arguments beyond the scientific domain to assess the morality of an action and guide decisions (IPBES 2022). However, differences in ethical perspectives and cognitive or emotional preferences, as well as the plurality of value systems, can generate different stances on conservation actions. Such differences can escalate toward conflicts (Redpath et al. 2013, Estévez et al. 2015, Crowley et al. 2017), which can be exacerbated under imperfect information and high uncertainty in conservation outcomes of these actions (Pollard et al. 2019). This is especially true for complex ecological systems involving many entities of nature. Note that we use the term *value* in the sense of “assigned value” (i.e., a value attributed to something) as opposed to “held value” (i.e., life goals and principles; cf. box 1).

Western conservation literature outlines three primary normative, ethical theories (box 1): consequentialist, deontological, and virtue. Under a consequentialist ethical framework, the framework likely most commonly considered by conservation managers, values are typically attributed to different entities of nature according to a given ethical perspective. The objective of such conservation approaches is then to maximize, reach, or maintain an acceptable overall value for all entities: Ecocentrism attributes intrinsic value to species and ecosystems, biocentrism attributes intrinsic value to living organisms, sentientism attributes intrinsic value to individuals that can experience consciousness beyond pain and pleasure, and anthropocentrism attributes intrinsic value to humans and instrumental value to other species (i.e., they are valuable insofar as they benefit humans; Latombe et al. 2022). Conservation targets could accordingly be set to maximize native biodiversity or its preservation (i.e., coevolved, natural communities; traditional conservation; Soulé 1985), maximize the benefits humans get from nature (new conservation, Kareiva and Marvier 2012, but see box 2 for a more nuanced description), or minimize animal suffering (conservation welfare; Beausoleil et al. 2018; see Latombe et al. 2022 for an in-depth discussion on how these perspectives can influence conservation decisions).

Box 1. **Definitions of concepts related to the valuation of nature and its entities**

**Anthropocentrism:** Ethical perspective that considers humans to be the sole or primary holder of intrinsic value, and therefore the concern of direct moral obligations. Nonhuman species are considered only in virtue of some relation they bear to humans (Norton 1984, Rolston 2003, Palmer et al. 2014).

**Assigned value:** A value attributed to something (in this case, mostly entities of nature) by someone, hereby expressing the importance they give to it compared to other things (Seymour et al. 2010).

**Biocentrism:** Ethical perspective considering all living beings as having intrinsic value and therefore the concern of direct moral obligations (Rolston 2003, Palmer et al. 2014).

**Consequentialism:** Normative ethical theory according to which an action morality is evaluated on the basis of its consequences (but see Sinnott-Armstrong, 2023 for different types of consequentialism).

**Deontology:** A normative ethical theory considering that “choices are morally required, forbidden, or permitted” (Alexander and Moore^ 2024^).

**Ecocentrism:** Ethical perspective considering that species, their assemblages and their functions, as well as more broadly ecosystems, rather than individuals, have intrinsic value and are the concern of direct moral obligations (Rolston 2003, Palmer et al. 2014).

**Entity of nature:** “Any concrete or abstract part of nature, encompassing, for example, species, landscapes, plants, animals, nature spirits and nature as a whole” (Lehnen et al. 2022). In this case, we focus on entities that “have morally significant interests” (Johnson 1983) and that also include animals, populations, species, and ecosystems.

**Held values:** Concepts or principles that human individuals deem important to them, underlying personal behavior, environmental beliefs, attitudes, and decisions (Seymour et al. 2010). Adhering to a specific ethical perspective is a held value.

**Intrinsic value:** Value expressed independently of any reference to people as valuers. Assigning an intrinsic value to entities of nature, including ecosystems or species, means acknowledging they are worth protecting as ends in and of themselves (IPBES 2022).

**Instrumental value:** Value given to an entity (individual or collective) on the basis of its utility and how it benefits another entity capable of attributing a value (in this case, humans). For example, dogs may have an instrumental value for herding sheep or as guard dogs (IPBES 2022).

**Moral psychology:** “The study of human thought and behavior in ethical contexts”; that is, their intuitive perception of the morality of actions and how they act on the basis of this perception (Doris et al. 2020).

**Normative ethical theory:** Theory defining what someone ought to do or, more generally, what is good (consequentialism), right (deontology) or virtuous (virtue ethics; Copp and Justin 2022).

**Principle of double effect:** Principle according to which it is permissible to cause harm as a consequence of an action that achieves positive consequences if this harm is not intended, but it is not permissible to cause harm intentionally, in particular as a means to achieve these positive consequences (McIntyre^ 2023^).

**Relational value:** Value reflecting “desirable, meaningful and reciprocal human relationships with nature and among people through nature” (IPBES 2022, but see Luque-Lora 2024 for a discussion on this concept and how it relates to instrumental and intrinsic values).

**Sentientism:** System considering sentient beings—that is, those with a conscience beyond perceiving pain and pleasure—as the concern of direct moral obligations (Rolston 2003, Palmer et al. 2014).

**Virtue ethics:** Ethical perspective that emphasizes the virtues or moral character as the reason for action (Hursthouse and Pettigrove^ 2022^).

Box 2. **Unresolved conflicts between conservation approaches.**

**Traditional versus new conservation:** Traditional conservation as defined by Soulé (1985) follows a consequentialist, ecocentric perspective, on the basis of the following normative postulates: diversity of organisms is good, ecological complexity is good, evolution is good, and biotic diversity has intrinsic value. Traditional conservation therefore aims at maximizing the preservation of these four quantities. By contrast, new conservation argues that maximizing the preservation of biodiversity can only be achieved through a utilitarian perspective (Kareiva and Marvier 2012). That is, even if they do not argue against the intrinsic value of biodiversity, proponents of new conservation consider that many stakeholders follow a utilitarian perspective, and that designing conservation actions aiming at preserving species or communities with instrumental values is the most effective approach for conserving biodiversity. This position has been heavily criticized by some proponents of traditional conservation, and, despite clarifications from new conservationists, the debate is ongoing (see, e.g., Kareiva and Marvier 2012, Soulé 2014, Doak et al. 2015, for different perspectives).

**Traditional versus compassionate conservation:** Compassionate conservation is a recent approach that is based on virtue ethics and promotes actions that stem from a compassionate attitude, following four tenets: do no harm, individuals matter, inclusivity (the value of an individual is independent from the context of the population, e.g., nativity, rarity, etc.), and peaceful coexistence (Ramp and Bekoff 2015, Wallach et al. 2018). Contrary to traditional conservation, as it is not a consequentialist approach, compassionate conservation cannot simply be seen as aiming to maximize these four elements. Instead, compassionate conservationists have established some stances on the basis of these tenets. For example, they oppose the killing of sentient animals by humans regardless of context, such as when an invasive species or a native predator threatens other native species because of anthropogenic change (Wallach et al. 2018). Compassionate conservation generated heated responses from traditional conservationists (e.g., Hampton et al. 2018, Driscoll and Watson 2019, Oommen et al. 2019), because such stances oppose the normative elements of traditional conservation mentioned above, and subsequent responses (e.g., Wallach et al. 2020) have not convinced opponents about the approach.

**Compassionate conservation versus conservation welfare:** Conservation welfare is a consequentialist, sentientist approach that aims to minimize animal suffering in conservation (Beausoleil et al. 2018). It therefore shares a focus on animal sentience with compassionate conservation (although compassionate conservationists have contradicted themselves in the literature, mentioning both the importance of considering individuals’ joy and pain, and their suffering, while at the same time advocating for considering all wildlife regardless of sentience; Wallach et al. 2018). Conservation welfare provides an objective criterion to determine the appropriateness of a conservation action (assuming suffering can be objectively measured). By contrast, compassionate conservation does not provide clear guidelines to determine what makes a conservation action guided by compassion beyond avoiding lethal actions, and how, for example, to resolve situations where lethal control would decrease overall suffering (Beausoleil 2020). Thought experiments depicting specific but hypothetical ecological situations were described by Rohwer and Marris (2019) as a basis for clarifying the stance of compassionate conservationists, but obtained no response, to our knowledge.

Under a deontological ethical framework, by contrast, actions are considered under the perspective of duties and rights. Conservation deontologists would reject the killing of animals to decrease overall suffering (a consequentialist perspective) if they consider that animals have inalienable rights to life (Korsgaard 2018, Bichel and Hart 2023). Finally, a virtue ethics framework advocates for actions that are driven by desirable human qualities (i.e., virtues; Hursthouse and Pettigrove^ 2022^), such as compassion for sentient animals (compassionate conservation; Wallach et al. 2018).

Because they are based on different premises, conservation approaches following different ethical frameworks have led to multiple but unresolved disputes in the scientific literature (box 2). In practice, conservation decisions need to consider both normative, ethical perspectives, and people's intuitive perceptions of the morality of actions (moral psychology; box 1), with the latter influenced by additional subjective, cognitive, and emotional factors. For example, the preference for immediate rewards over avoidance of long-term or distant costs is a highly context-dependent psychological trait (Critchfield and Kollins 2001, van der Wal et al. 2013), which has implications for conservation: People are averse to the sacrifices of concrete, immediate benefits that contribute to long-term and more abstract climate change (Swim et al. 2009). People tend to be less cooperative with conservation actions if there is uncertainty in their outcome, in the supporting funding, or in the extent of community support, and these types of uncertainty can affect the public's intention to cooperate (Pollard et al. 2019). Similarly, cultural preferences, species charisma, and relationships with entities of nature (relational value; box 1) can influence decision-making (Díaz et al. 2018, Lehnen et al. 2022). Some traditional cultural practices can threaten entities of nature, and the ethical value of such cultural practices can collide with the value of the entities they affect (Dickman et al. 2015). The interplay between ethical views and subjective or intersubjective preferences can also generate personal dilemmas (internal conflicts)—for example, when species are affected differently by different conservation measures or when normative perspectives conflict with a cognitive or emotional preferences (rationality versus emotion). Such external and internal conflicts can complicate decision-making (Peterson et al. 2005).

This suggests a tool is needed for conservation practitioners to systematically compare the positions of stakeholders with different worldviews but also to critically examine their own positions and how they vary with context. Such a tool will promote communication between parties, identify points of divergence or agreement, and make conservation decisions ethically more robust.

## The trolley problem thought experiment as a tool to capture the diversity of conservation stances

Metaphors are often used to conceptualize issues and clarify moral reasoning (Johnson 2014). They can be used to create thought experiments simplifying real, complex situations and thus to explore how moral intuitions naturally emerge or to elaborate more robust moral stances under normative theories (Lakoff and Johnson 1999, Haidt 2001, Singer 2005). Metaphors can therefore be used to solve, or at least identify the origin of, disagreements and conflicts in conservation. For example, thought experiments have been proposed to clarify the ethical system of compassionate conservationists (Rohwer and Marris 2019).

The trolley problem (Foot 1967, Thomson 1976) is a well-known thought experiment to explore ethical dilemmas when both an action and an absence of action will necessarily affect humans negatively; it has been used both in arguments for different normative ethics (e.g., Singer 2005) and in moral psychology experiments (e.g., Greene et al. 2001, Navarrete et al. 2012). In its general formulation, a carriage of a tram (hereafter, a *trolley*) is out of control and cannot be steered or slowed down. It is heading toward five people who are stuck to the track and will be killed on impact. The choice is whether to pull a lever that will divert the trolley onto another track, where another person is stuck. The (forced) choice is to act and save five but kill one other or to do nothing and let five people die. Should the lever be pulled?

**Figure 1.**
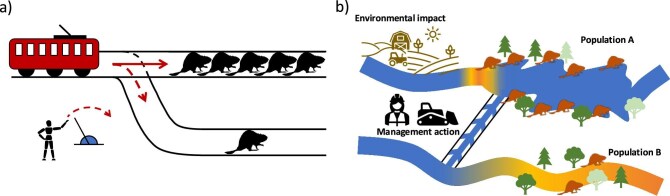
The trolley problem applied to conservation. (a) In the original version, entities of nature are attributed the same intrinsic or instrumental value—in this case, individuals of the same species. The tracks represent two conservation management options: The upper tracks are a laissez-faire option that will lead to many individuals being harmed, whereas the lower tracks represent the implementation of a management action that would lead to fewer individuals being harmed, but those would not have been harmed without the action. Note that the individuals on the two tracks belong to different populations and that the population on the second track is not a subset of the first one. (b) A framing of the trolley problem in a conservation context. A large population of beavers is affected by anthropogenic activity that cannot be prevented. Diverting water from a nearby river would save them but would lead to the death of a distinct, smaller beaver population.

The trolley problem can be adapted for conservation by replacing the humans on the tracks with entities of nature. The trolley therefore becomes a metaphor for an environmental change that will cause harm (figure 1a). Different conservation approaches put different emphasis on individuals, populations, species, ecosystems, function, or even genes (box 2). In the present article, we mostly illustrate this thought experiment with individual animals and animal populations, sometimes from different species, because they are more straightforward to evoke as being physically present on tracks. Nonetheless, the reasoning can be extended to different taxonomic groups (e.g., plants) or to ecosystems that are also frequently targeted by conservation actions and of which individuals and populations are the fundamental building block. We also mostly consider situations with two options, although in practice, multiple options may be available, sometimes in combination, with different outcomes. The trolley problem metaphor is crude (cf. Bauman et al. 2014), but this simplicity allows for trade-offs in conservation to be systematically explored, thereby helping decision-making under moral quandaries.

Consider a scenario where a lake is drying out (figure 1b). If the lake is lost, a large number of beavers will die (provided they cannot move to an alternative habitat). A hydrologist proposes diverting a nearby river to feed the lake. However, if the river is diverted, another beaver population will lose its habitat and die. Importantly, in this version of the trolley problem, the environmental change is of the same nature: In either case, a population would disappear because of the lack of water, and the two populations are distinct. Should the interbasin water transfer scheme be built? In the thought experiment, the trolley is therefore a metaphor for the lack of water, which is the factor potentially affecting the two populations.

In the following, we develop variations of the trolley problem to explore key concepts in different conservation contexts (tables 1–3). We do not seek to discuss the validity of individual conservation approaches or moral stances under conservation trolley problem variations, nor do we intend to recommend specific sets of choices. Rather, we seek to break down conservation issues into their core elements and explore how they will potentially influence moral intuitions or reasoning in conservation contexts in order to identify where conflicts or moral dilemmas may arise. The conservation trolley problem can therefore be seen as a visual aid to generate a catalogue of concepts that can affect moral judgment and play a role in conservation decisions. In particular, we develop variations covering three main concepts that are likely to influence conservation decisions (with different levels of relevance for comparing normative ethical theories or for discussing subjective individual stances on conservation issues): uncertainty and risk about the outcome of an action and unforeseen consequences (see table 1 and the “Uncertainty, risk, and unforeseen consequences” section); asymmetry between the tracks (in numbers, in victims, in impacts, and spatiotemporal; see table 2 and the “Variations of the conservation trolley problem with conservation options involving asymmetry between tracks” section); and causal effects and the involvement and responsibility of stakeholders (see table 3 and the “Variations of the conservation trolley problem involving causal effects and the involvement and responsibility of stakeholders” section).

**Table 1.**
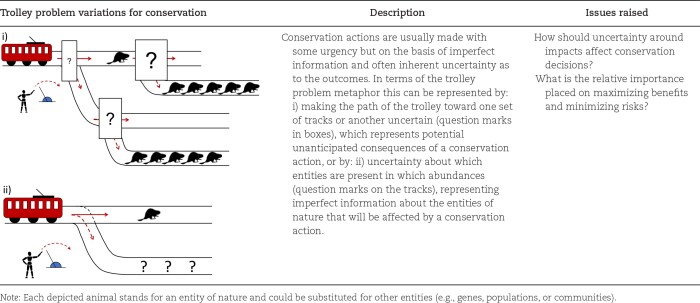
Variations of the trolley problem with conservation analogues and insights for decision-makers, focusing on issues of uncertainty and unforeseen consequences variation.

**Table 2.**
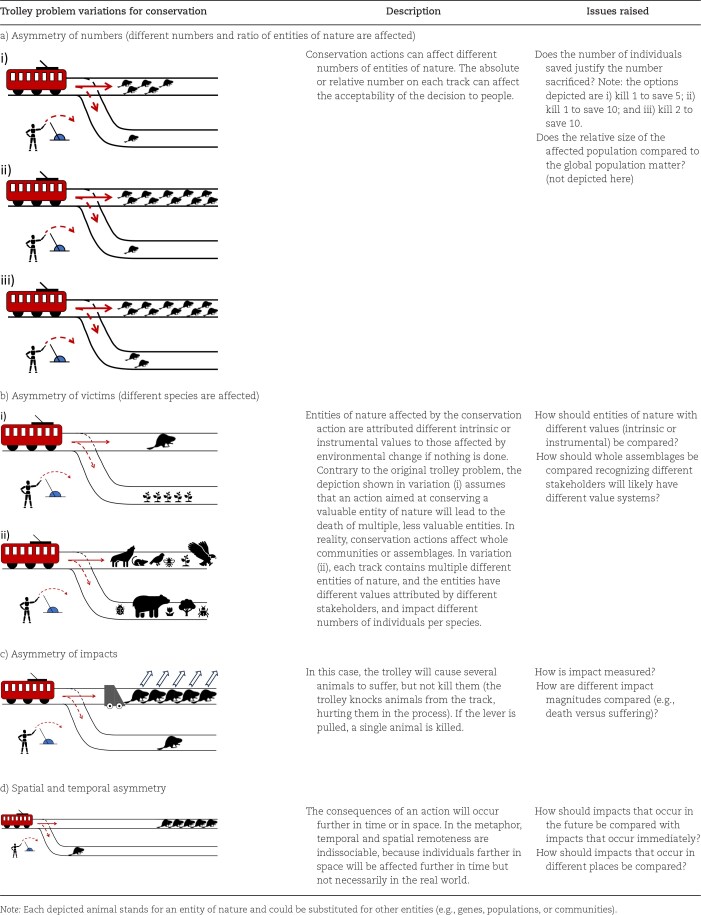
Variations of the trolley problem with conservation analogues and insights for decision-makers, focusing on issues of asymmetry between tracks.

**Table 3.**
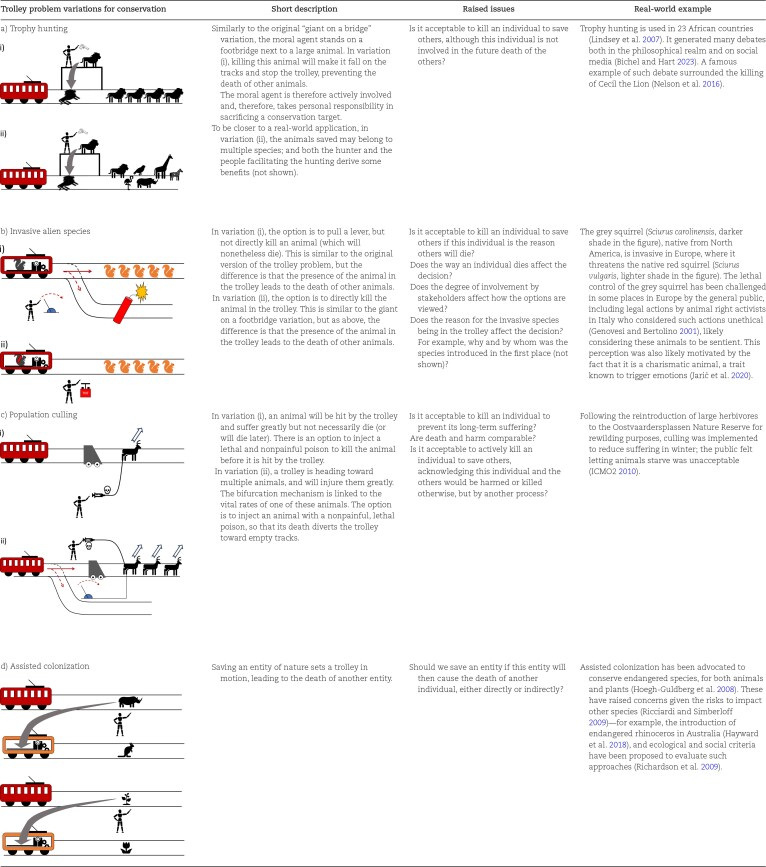
List of variations of the trolley problem for real-world conservation issues involving different relationships between the presence of an individual and the death of others, and different levels of involvement of stakeholders.

## Uncertainty, risk, and unforeseen consequences

In the original trolley problem and in the different variations below, there is a clear cause (pulling the lever) and effect (one individual dies rather than five). However, this determinism is rarely clear in conservation. In reality, uncertainty and risk will interact with asymmetry to generate additional dilemmas and conflicts. This is especially true if the benefits of the action have been overestimated or when the costs in the business as usual scenario (not pulling the lever) have been underestimated. Uncertainty and risk can be added to the trolley problem by adding probabilistic branching on the tracks representing the lack of information about those probabilities or about the abundance and number of species on a track (table 1).

Uncertainty is inherent to conservation and hard to reduce or even to quantify (Canessa et al. 2016). Acquiring new information is also not always guaranteed to reduce uncertainty (Canessa et al. 2015). Rationally, there are multiple ways of approaching risky dilemmas, such as taking the action that ensures the least negative outcome, that ensures the best average outcome, or that has the potential for the most positive outcome. These approaches can be compared in normative perspectives. However, from a moral psychology perspective, agents tend to use different decision-making strategies if outcomes are uncertain, relying less on consequentialist or utilitarian approaches (Kortenk Amp and Moore 2014). If the decision-makers are risk averse, as is often the case, then business as usual might be favored even if it represents the least favorable option from a consequentialist perspective (Canessa et al. 2020). In conservation, uncertainty has been shown to be a major deterrent for the implementation of specific actions owing to the fear of failure and anticipating potential negative effects of these very actions. This fear of failure is exacerbated in the case of potential catastrophic impacts—for example, losing the last remaining population of a species (Meek et al. 2015). We can therefore expect uncertainty to potentially have important effects on how people perceive the morality of the different trolley problem variations presented below. To which extent and how these effects will vary with the moral and psychological aspects related to each variation are unknown and have been, to our knowledge, seldom examined.

Fear of failure can be reduced by increasing the sharing of information among stakeholders, providing better decision-support tools, promoting collaboration, and building consensus expectations, but also more consistently reporting project failures in order to learn from mistakes (Catalano et al. 2019). However, as in the metaphor of Chesterton's fence, there can also be value in being inherently cautious about change (i.e., don't remove a fence if you don't know why someone put it up in the first place), and the level of uncertainty and of impact will likely influence one's attitude toward such conservation situations.

## Variations of the conservation trolley problem with conservation options involving asymmetry between tracks

In this section, we provide a suite of variations of the conservation trolley problem in which the impacts or the entities of nature are different between the tracks. The tracks often differ in two aspects that oppose each other, potentially generating dilemmas or conflicts.

### Asymmetry of numbers

In the original human-based trolley problem, one should always save the larger number of people under utilitarianism, and the numbers should not matter for deontological considerations. However, in a moral psychology context, it has been shown that the ratio of the number of people on the different tracks can influence the decision to pull the lever (Nakamura 2012). Because different conservation issues inherently weigh different population sizes against each other, similar considerations may be important for nonhuman entities of nature (table 2a). For example, in trophy hunting (see table 3a and the “Trophy hunting variations” section), one or few individuals may be killed by a wealthy hunter to generate revenues that will contribute to saving many other individuals. By contrast, lethal control of a population of an invasive alien species is likely to result in the death of many individuals, and both the absolute and relative sizes of populations on the tracks will differ from the trophy hunting case. The number of entities of nature on the respective tracks of the conservation trolley problem can help us determine whether the differences in numbers may or should influence conservation decisions.

The ratio between the size of the affected and global populations may also be important. Under biocentric and sentientist perspectives, which give intrinsic value to individuals, the absolute size of the affected population will likely determine whether there is a dilemma. Under the ecocentric perspective, which gives intrinsic value to species and populations, the relative size of the affected population with respect to the total local or global population of the species, representing the threat on the population or species, will likely determine whether there is a dilemma. For example, the eradication of a large invasive population of the house mouse (*Mus musculus*) from an island might be considered justified if it saves a small population of a globally rare species of seabird. In this case, there is variation in both the numbers of animals affected on the island and the relative global abundances of the two species.

### Asymmetry of victims

As in the mouse-infested island case above, conservation actions usually affect different species, which can even belong to different kingdoms. In such cases, conflicts and dilemmas are likely if species are attributed different intrinsic, instrumental, or relational values or are considered to have different rights but differ in population size (table 2b). For example, trophy hunting (see table 3a and the “Trophy hunting variations” section) targets charismatic species that are often attributed high values. Assisted colonization (see table 3d and the “Assisted colonization variations” section) will also target species of high value, with potential deleterious effects on other species. From a consequentialist perspective, affecting a single, highly valuable individual (or more generally a highly valuable small population) could be considered equivalent to affecting many less valuable individuals (and by extension a less valuable larger population), if we consider these values can be added up (Latombe et al. 2022, Jarić et al. 2024). This equivalence will depend on the different elements (be they ecological, ethical, subjective) that determine the value attributed to an entity of nature, such as their sentience, rarity, charisma, cultural or economic importance, or endemicity (IPBES 2022). Asymmetry of victims can be represented in the trolley problem by considering different species on the tracks to explore such considerations.

### Asymmetry of impacts

Conservation actions might aim at individual deaths or population extirpation but might also cause or prevent the deterioration of the physical state and well-being of individuals. Pollution and changes in land cover, food availability, hydrology, or temperature affect the behavior (e.g., sleep patterns, activity level, risk taking) and the physiology (e.g., metabolic rate) of animals (Killen et al. 2013, Raap et al. 2015). The dilemma arises if many individuals endure nonlethal impacts in one case and few individuals would die or endure a higher impact in the other. Nonlethal impacts, such as food deprivation, have led to public reactions and changes in conservation actions (ICMO2 2010). In the trolley metaphor, nonlethal impacts can be represented by the trolley injuring but not killing the individuals on the tracks (table 2c). Doing so could help clarify how normative ethical theories weigh nonlethal versus lethal impacts (see, e.g., Driscoll and Watson 2019), although the cause of the impacts is also likely to matter (see table 3 and the “Variations of the conservation trolley problem involving causal effects and the involvement and responsibility of stakeholders” section below).

### Temporal and spatial asymmetry

Temporal and spatial discounting is a well-known cognitive trait that means people value the consequences (reward or cost) of an action less if the consequences occur far away or a long time in the future (Green et al. 1997, Critchfield and Kollins 2001, Perrings and Hannon 2001). Discounting rates can affect environmental management. For example, differences in discounting rates between human populations from different countries or states regarding the value of the natural environment can lead to differences in investment to implement environmental policies such as greenhouse gas emissions (Carson and Roth Tran 2009). To our knowledge, this concept has been seldom applied to examining how people may value impacts on entities of nature outside of economic considerations. Temporal and spatial asymmetry is also important from normative perspectives. For example, should the fate of future individuals, who are not born yet, influence conservation decisions? Should we aim to prevent future generations from starving by culling individuals that are alive today? Similarly, should individuals that will not be born because of the implementation or absence of a conservation action (including sterilization campaigns) be valued and influence conservation decisions? In other words, is it morally wrong to prevent individuals from living if they never existed in the first place? Temporal and spatial asymmetry can be represented using the trolley problem metaphor when entities are far away from the junction on the tracks (table 2d).

## Variations of the conservation trolley problem involving causal effects and the involvement and responsibility of stakeholders

Asymmetry and uncertainty variations capture important considerations met by conservation managers facing environmental issues and are important both for defining and comparing normative theories and for understanding moral psychology in conservation. However, causal relationships among the presence of an entity, the death of another, and the type of action taken are seldom explicitly considered in conservation decisions. By contrast, variations of the original trolley problem have been widely used to examine how stakeholder involvement and causality are important for ethical decisions involving humans. These variations have been used to explore normative theories—for example, for the principle of double effect (see below)—and to understand ethical decision-making in moral psychology.

### Giant victim variations of the original trolley problem

**Figure 2.**
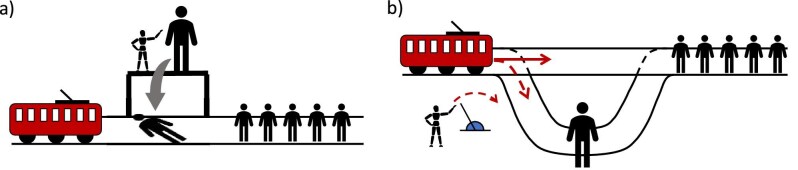
Two variations of the trolley problem in which the intentionality of actions differ. (a) The original giant on a footbridge variation from Thomson (1976), in which the trolley can be stopped by pushing a giant onto the tracks; contrary to a normal person in the classical trolley problem, the giant, because of its size, stops the trolley, thereby saving any persons that are farther down on the tracks. (b) The giant on a loop variation, in which the giant is stuck on a loop to which the trolley can be diverted, therefore stopping the trolley and saving the five other people (Thomson 1985, Singer 2005). In both cases, the giant is used as a means to prevent the death of other people and is not a consequence of saving others, as in the original trolley problem. According to the principle of double effects, the two variations should be morally equivalent. Nonetheless, the action differs in the two variations (pushing someone versus pulling a lever). If normative ethical perspectives recommended different conclusions, that would refute the principle of double effect and put the emphasis on the nature of the action. It has been shown that people often make different decisions in these two variations (Awad et al. 2020), which may be due to emotional connections to the action to be taken.

Although initially outlined by Foot (1967), the trolley problem only gained prominence through Thomson's (1976) famous *fat man variation* (hereafter termed more neutrally the *giant on a footbridge variation*; figure 2a). In this variation, there is only one set of tracks, to which five people are stuck. A person on a footbridge next to a giant realizes that pushing the giant onto the tracks will stop the trolley and save the five people, killing the giant. Should they push the giant?

The comparison between this thought experiment and the original trolley problem has significant implications for understanding ethical decision-making, both for normative theories and for moral psychology. Considerations of causal links between the death of individuals and the survival of others and the implication of stakeholders also apply to decision on conservation practice, such as trophy hunting (table 3a), lethal management of invasive alien species (table 3b), population culling (table 3c), or assisted colonization (table 3d), which imply different levels of involvement from stakeholders and different causal relationships between the presence of individuals and populations and the death or suffering of others, generating conflicts among stakeholders but also conservationists (Baynham-Herd et al. 2018, Wallach et al. 2018, Driscoll and Watson 2019). Before presenting these conservation variations, it is important to illustrate the ethical and psychological issues raised by the giant on a footbridge problem, to better understand the importance and consequences of seemingly small differences.

The giant on a footbridge variation has been discussed from a normative theory perspective (e.g., Thomson 2008, FitzPatrick 2009). In particular, the principle of double effect has been used to defend the action of pulling the lever in the original trolley problem but not pushing the giant. According to this principle, it is morally unacceptable to intentionally cause a negative effect as a means to achieve a positive one—that is, to push and kill the giant as a means to save five people. Conversely, causing unintended harm while attempting a good deed, as in the original trolley problem, where pulling a lever diverts the trolley to save five but inadvertently kills one, is viewed more favorably (McIntyre^ 2023^). These variations were taken further by the giant on a loop variation (Thomson 1985, Singer 2005). A lever can be pulled that would divert the trolley to a loop on which the giant is attached, therefore stopping the trolley but killing the giant (figure 2b). This last variation was used to show how small differences between variations can affect whether actions are viewed as moral or not, and it is still debated whether killing the giant in the loop variation is a means or a consequence of saving the other five (Bruers and Braeckman 2014, Kaufman 2016). We therefore expect small variations in how entities of nature are used as means or how they may affect other entities to raise similar ethical concerns in conservation.

These variations have also been used to explore people's moral psychology. In the original trolley problem, most people (around 90% across multiple studies) act consistently with a utilitarian approach (box 1) and sacrifice the single person (Navarrete et al. 2012), whereas most people would refuse to push the giant (Greene et al. 2001, Singer 2005, Awad et al. 2020). A large international survey has shown that people globally consider that sacrificing one is less acceptable in the giant on a loop than in the original variation but is more acceptable than in the giant on a footbridge variation (with country-dependent variations; Awad et al. 2020). The results from functional magnetic resonance imagery suggest that there is a stronger emotional response to the idea of killing a person by pushing them than the idea of killing someone by pulling a lever (Greene et al. 2001). We therefore expect emotional responses to differ regarding different types of conservation strategies, in particular between lethal and nonlethal ones.

In the following sections, we develop similar variations for conservation situations involving different levels of implication from stakeholders and different relationships between entities of nature on the tracks to explore how these may influence conservation decisions.

### Trophy hunting variations

Trophy hunting for conservation—allowing to hunt charismatic animals to fund conservation of entire populations and protected areas—is a conservation practice that can contribute to preserving endangered species, but that has also generated ethical, social, and biological problems (Lindsey et al. 2007). Trophy hunting can be illustrated using a conservation analogue of the giant on a footbridge to explore whether it is considered unethical relative to the base version of the conservation trolley problem (table 3a): The option is to push an animal on the tracks (i.e., kill it) to save several animals of the same or a different species farther down the tracks. Another variation more in line with trophy hunting would be not to directly kill an animal but to allow someone else to do it, which may decrease the impression of responsibility and potentially change the acceptability of the action. Further subtleties can be included, such as the motivation of the person who would kill the animal (Macdonald et al. 2016), the charismatic attributes of the animal, the degree to which funds raised through trophy hunting go directly into conservation or to those who facilitate the hunting, and the degree to which any of the actions causes suffering, although these considerations may be harder to explicitly depict in the thought experiment.

### Invasive alien species variations

Invasive alien species are among the main threats to global biodiversity (Bellard et al. 2016, Maxwell et al. 2016, Roy et al. 2024). The eradication of invasive alien species (which typically involves the lethal control of invasive alien animals and populations) has been found to be among the most effective conservation interventions (Jones et al. 2016, Langhammer et al. 2024, Sankaran et al. 2024). However, lethal control of invasive alien species has been criticized for being unethical when applied to sentient beings, particularly vertebrates (Wallach et al. 2018, 2020), leading to conflicts between stakeholders (Crowley et al. 2017). The causes for such conflicts are often not obvious and may be due to concepts of asymmetry and uncertainty but also to involving actions with the direct intention of killing animals, such as trapping, hunting, or poisoning (Crowley et al. 2017), sharing similarities with trophy hunting. The main differences to the trophy hunting variation are the different number of individuals affected and the threat posed by invasive alien species individuals to individuals of native species (or native entities of nature), therefore warranting a distinct variation to examine differences in normative views and in people's stances between invasive alien species lethal control and trophy hunting.

For a better understanding of this causal relationship, we suggest two variations of the conservation trolley problem (table 3b). In both variations, an individual of the invasive alien species sets the trolley in motion—for example, after its presence is recorded from a weighing scale. In the first variation, the option is to pull the lever and send the trolley on a path that will kill the individual of the invasive alien species inside it. This represents environmental management that benefits the native species but is detrimental to the invasive alien species. In the second variation, the option is to push a button that will directly kill the invasive alien species entity and stop the trolley. These two variations entail different levels of involvement of the stakeholder in how the death of the invasive alien species is a side effect or a means to allow the other individuals to survive. It is also possible to distinguish multiple reasons for the invasive alien species being on the trolley, to reflect different pathways of introduction, intentional (e.g., introduced for hunting) or unintentional (e.g., animals that moved along a human-built corridor; Faulkner et al. 2024). Additional considerations may be important to consider and depicted in the trolley problem—for example, if the invasive taxon is endangered in its native range (e.g., the sea lamprey *Petromyzon marinus*, Guo et al. 2017, and other examples, Tedeschi et al. 2025).

### Population culling variations

Population culling is used in conservation for various reasons—for example, to prevent overconsumption and starvation through overpopulation, to decrease predation pressure, or to stop the spread of epizootics (VerCauteren et al. 2011; e.g., Hervieux et al. 2014, Wilson et al. 2015, Martel et al. 2020). Culling raises inherent ethical concerns (Wallach et al. 2018) and has generated opposition from the public (e.g., ICMO2 2010, Nugent et al. 2011). To conceptualize the issue and explore the causes of these conflicts, we propose two variations of the conservation trolley problem (table 3a). As in the asymmetry of impact variation, we assume that the trolley will not kill an individual on impact but will injure it greatly, inflicting high levels of suffering. In the first variation, the option is to inject the animal with a lethal, quick, and nonpainful poison. This variation explores the morality of killing a—potentially sentient—living being to prevent its future suffering. In the second variation, injecting the animal would save other animals from suffering (in the trolley metaphor, the lever is somehow attached to the animal's vitals; injecting the animal kills the animal but puts the trolley onto an empty track). The second variation is similar to a situation when the presence of an invasive alien species would not result in the death of native individuals but would degrade the quality of life of both native and invasive species. The difference for population culling is that entities are indistinguishable from each other, whereas for invasive alien species, it is the introduction of specific entities that generates suffering.

### Assisted colonization variations

Assisted colonization the translocation of individuals or populations of endangered species outside their indigenous range to prevent their extinction, can be contentious because of the threat they can cause to species native to the introduced range (Hoegh-Guldberg et al. 2008, Ricciardi and Simberloff 2009). The consequences of translocation can be explored using a variation of the conservation trolley problem (table 3d). The animal or entity of nature at threat from being killed is moved out of harm's way but onto another trolley. This second trolley is set in motion by the weight of the animal and starts moving toward individuals of another species or other entities of nature that will be killed or injured by the second trolley. Should the animal be moved?

## Discussion and conclusions

We presented variations of the trolley problem for conservation to clarify and compare complex situations, as illustrative thought experiments (*sensu* Brun 2017). These variations can also be used in a heuristic fashion (*sensu* Brun 2017) to identify contexts likely to create ethical dilemmas, conflicts between stakeholders, or conflicts between normative theories (see box 2 and supplemental file S1). Using the trolley problem to compare trophy hunting with the lethal control of invasive alien species (table 3a, b), we can see that they share a direct involvement of stakeholders (there is a lethal action in both cases) but differ in the relationship between the individuals being killed and the other individuals; they likely differ in the number of individuals that are killed; they likely differ in the relative value of the entities of nature affected (notably, trophy hunting often targets charismatic animals with high, local cultural value, whereas invasive species range from those that are highly desired to those that are highly despised; Jarić et al. 2020); and they differ in the magnitude and type of uncertainty linked to the action, with uncertainty potentially lying in the efficacy of the approach for invasive species control and in how money will be invested in conservation for trophy hunting and therefore how beneficial effects will be for other species. The trolley problem allows a common depiction to explore which of these variations are likely to lead to disagreements and dilemmas.

Conservation actions can affect humans positively (e.g., by generating income) and negatively (e.g., by restricting activities or costing money). As the value attributed to humans likely differs from that attributed to nonhuman entities of nature, it will be important to in addition, consider affected stakeholders in future thought experiments. The loss of different entities of nature with incommensurable value to humans can generate choices and dilemmas that are very difficult to resolve when weighed against commensurable quantities, such as costs of management actions or profits (Daw et al. 2015; see supplemental file S2 for more details). It is similarly important to explore different levels of stakeholder involvement and causal relationships between entities of nature's existence and death, and combining these with asymmetry and uncertainty variations (see supplemental file S1 for an example for invasive alien species). Doing so may prove especially useful to break down problems typified by multiple elements interacting in a complex system that do not have a single tractable solution—that is, wicked problems (Game et al. 2014, Woodford et al. 2016).

The trolley problem metaphor is, of course, not appropriate for all conservation issues, nor does it capture the full complexity of most conservation situations. The metaphor focuses on a crisis situation (an urgent choice needs to be made). By contrast, conservation decision-makers are often faced with choices concerning proactive actions (e.g., prevention) or long-term planning. The trolley problem clearly demonstrates, however, that postponing conservation decisions or actions can have important and irreversible consequences (e.g., Naujokaitis-Lewis et al. 2018). In addition, the importance of the biodiversity crisis may warrant conveying a sentiment of urgency, just as if the trolley were moving toward the junction. Entities of nature also encompass multiple concepts, from individuals to collectives: It may be less intuitive to apply the trolley problem to species than to individuals or populations, and it is probably even less intuitive to compare impacts on individuals with impacts on species, communities, or ecosystems, although the latter are central to conservation (Luque-Lora 2023). As we expect trolley problems to be more easily applicable for relatively simple systems with a limited number of options and species involved, it may have limitations when examining dilemmas with many species and habitats, for example. The conservation trolley problem also lacks important conservation aspects such as the concepts of naturalness of conservation actions (is an action implemented by humans disconnected from nature?), and the different roles humans can take with respect to nature (observer, participant, partner, explorer, steward or manager; Wienhues et al. 2023), although these concepts can be, at least partly, related to the involvement of stakeholders in the trolley problem. Finally, the trolley problem presents a forced choice among mutually exclusive actions, whereas real-world conservation often relies on combinations of sometimes related management actions. Because of all the simplifications it makes, the trolley problem metaphor should not be used in isolation as a conservation decision tool, nor can it be expected to solve conflicts and disagreements on its own. Rather, it should be seen as a tool to complement other existing ones in such situations. Conservation decisions will still require a range of approaches to compare different alternatives (e.g., cost–benefit analyses or multiple-choice decision analyses), and to capture stakeholders’ opinions and perceptions (e.g., guided or unguided interviews or group facilitation methods; see Bennett et al.^ 2017^ for a list of social science approaches to conservation). In this context, the trolley problem may allow decision-makers to better capture diverging world views from different stakeholders (IPBES 2022) but can also be a tool for stakeholders to reflect on.

Nonetheless, the trolley problem is a versatile tool to capture future impacts of different conservation actions (or a lack thereof), and generate a wide variety of scenarios combining different variations. Trolley problem variations provide a robust foundation to develop nuanced and context-specific metaphors, explore and communicate management situations that can generate conflicts, and ultimately contribute to better informed and ethically sound conservation practices. Specifically, we recommend the exploration of the three following topics. First, different conservation approaches based on normative theories should clarify their positions, especially new and compassionate conservation, which have generated heated but unresolved debates (box 2). The normative postulates of traditional conservation provide clear objectives to assess the value of conservation actions. New conservation does not provide such postulates and, instead, defines practical statements (Kareiva and Marvier 2012), which do not allow the same kind of assessment. The postulates of compassionate conservation (Wallach et al. 2018) are also currently too vague to unambiguously and objectively guide conservation choices by themselves. The trolley problem metaphor could be used to derive a suite of variations for which a conservation approach would define an appropriate choice. This could then be used to better identify clear normative postulates.

Second, variations and thought experiments that explicitly incorporate additional concepts such as naturalness should be developed. A trolley may be an appropriate metaphor for the construction of a train line or a highway, but other metaphors could be more appropriate in different contexts. However, the strength of a framework is often in its simplicity, which offers some common ground to discuss, and using too many metaphors may be counterproductive. In the present article, we recommend using the trolley problem as the base framework to develop variations and to use other context-specific metaphors to ease communication afterward.

And third, the conservation trolley problem metaphor and other conservation thought experiments could be applied to moral psychology—for example, through surveys—to better understand people's attitudes toward nature conservation. In this context, it will be important to explore how different stakeholder characteristics (e.g., gender, economic status, and cultural background), roles (e.g., scientists, policymakers, managers, and users), and training in natural and social sciences (in particular against unconscious biases) may affect their decisions.

## Supplementary Material

biaf052_Supplemental_File

## Data Availability

No new data were generated or analysed in support of this research.

## Author Biography

Guillaume Latombe (glatombe@ed.ac.uk) is affiliated with the Institute of Ecology and Evolution at The University of Edinburgh, in Edinburgh, Scotland, in the United Kingdom. Ugo Arbieu, Franck Courchamp, and Ivan Jarić are affiliated with the Université Paris-Saclay, CNRS, AgroParisTech, Ecologie Société Evolution, in Gif-sur-Yvette, France. Sven Bacher is affiliated with the Department of Biology at the University of Fribourg, in Fribourg, Switzerland. Stefano Canessa is affiliated with the Division of Conservation Biology at the Institute of Ecology and Evolution at the University of Bern, in Bern, Switzerland, and with the Dipartimento di Scienze e Politiche Ambientali, Universitá degli Studi di Milano, in Milan, Italy. Stefan Dullinger is affiliated with the Division of Biodiversity Dynamics and Conservation in the Department of Botany and Biodiversity Research at the University of Vienna, in Vienna, Austria. Franz Essl, Michael Glaser, Bernd Lenzner, and Anna Schertler are affiliated with the Division of BioInvasions, Global Change, and Macroecology in the Department of Botany and Biodiversity Research at the University of Vienna, in Vienna, Austria. Ivan Jarić is also affiliated with the Biology Centre of the Czech Academy of Sciences, Institute of Hydrobiology, in České Budějovice, in the Czech Republic. Anna Schertler is also affiliated with the Vienna Doctoral School of Ecology and Evolution, at the University of Vienna, in Vienna, Austria. John R. U. Wilson is affiliated with the South African National Biodiversity Institute, Kirstenbosch Research Centre, in Cape Town, and with the Centre for Invasion Biology, Department of Botany and Zoology, Stellenbosch University, in Stellenbosch, South Africa.
