# Supplementary material for: Clarifying ethical stances in conservation: a trolley problem thought experiment
Source: Bioscience. 2025 Jun 17;75(9):722–36. doi: 10.1093/biosci/biaf052 (PMC12412296; doi:10.1093/biosci/biaf052)
Supplement: biaf052_Supplemental_File [file biaf052_supplemental_file.docx]

Supplementary material for: “Ethical dilemma in conservation: a trolley problem thought experiment”

Guillaume Latombe, Ugo Arbieu, Sven Bacher, Stefano Canessa, Franck Courchamp, Stefan Dullinger, Franz Essl, Michael Glaser, Ivan Jarić, Bernd Lenzner, Anna Schertler, John R. U. Wilson

Appendix S1. Avenues to combine trolley problem variations and apply it to conservation issues

Conservation partners and stakeholders or proponents of different conservation approaches driven by normative theories may disagree over a conservation action for different reasons, which can be difficult to pinpoint exactly, leading to further conflict and frustration. Here we suggest an approach to combine the different variations of the trolley problem presented in this paper in a systematic fashion, to explore which aspect of the action generates opposition. The trolley framing by itself will not solve conflicts, generate practical actions or create consensus; rather, it is meant as a diagnostic step to facilitate dialogue and clarify potential for conflict resolution. In addition, framing the issue using the trolley metaphor may present the issue to stakeholders in a different light, raise understanding of other stakeholders’ value systems, leading to a change in their stance or how strongly they feel about it. In addition, other combinations of variations may be appropriate for specific situations. Ideally, this process would be embedded in a broader structure for rational decision making, such as systematic conservation planning, structured decision making, or open standards for the practice of conservation (Schwartz et al. 2018).

**Framing.** The first step is to clearly define the action being discussed, as well as its spatial and temporal scale and scope. One should identify the decision makers, stakeholders and partners involved. The objective(s) of the action should be absolutely clear to all involved: these define what goes on the tracks of the trolley scenarios, that is, the entities being considered and how they are represented/quantified. A model of the system is necessary to fill in the problem with the different outcomes, that is, the individuals/entities that die in the different scenarios, and any other relevant objectives.

**Variations.** The original trolley problem is unlikely to capture the realistic conditions of the real-world problem at hand. Therefore, the group should develop a set of variations to explore the influence of different aspects. It is important here to strike a balance between realism and abstraction: overly complicated problems may become less and less informative as a diagnostic. If divergences or conflicts remain when the most complex version of one element is built, it indicates that other elements are important, and one can move to the next (for example, from involvement to uncertainty).

***Involvement.*** Develop a set of trolley problem variations with increasing direct involvement of stakeholders in the death of the entity, until most elements from the real situation are captured (Figure S1a). The last variation of the phase 1 of the framework should be the closest to the concrete situation in terms of causal relationships between species’ life and death and of stakeholder involvement. For example, lethal management of invasive species is often recommended in early stages of invasion, when eradication is still possible (Pluess et al. 2012), usually implying active killing of individuals. In the context of invasive species management, one can therefore investigate if stakeholders have different stances about the indirect and direct killing of individuals, which would correspond to environmental management vs. lethal approaches, and the impacts these individuals have on others (Figure S1a).

***Asymmetry*.** To explore additional components, one should progressively add different species that may be affected by the choice and discussing their values (asymmetry of victims), different types of impacts including lethal and non-lethal (asymmetry of impacts), and different spatial and temporal scales at which impacts will be realized (spatio-temporal asymmetry) (Figure S1b). For example, will lethal trapping of invasive species incur by-catch of native species, including common or endangered ones? Would the removal of invasive species cause suffering in the immediate future, but avoid larger impacts in the longer term, or at a larger scale?

***Uncertainty.*** Given the nature of conservation problems, capturing some level of uncertainty will likely always be necessary. In this case, the trolley representation might be modified to reflect a decision tree (Canessa et al. 2016), for example by choosing a small set of scenarios with possible (uncertain) outcomes to discuss, represented by different tracks (Figure S1b). This uncertainty should ideally come directly from the model of the system used to represent the outcomes of the action in the original problem description. For example, one could represent different velocities of spread by the target invasive (represented by different levels of impact on native species) or different levels of by catch by untested removal methods. The different scenarios can initially be described verbally, and if needed then associated with their different probability of occurrence.

***Combining variations.*** Finally, if no change of opinion still arises, we recommend combining these variations in phase 3 (Figure S1c). How to combine these variations will likely be context dependent.

**Discussion.** Depending on the sensitivities, one may need to adopt different facilitation methods to lead stakeholders and groups through the process. Ideally, best practice methods for dealing with groups, including a careful composition of groups. To avoid dominance and power dynamics, as well as common heuristics such as anchoring, discussions should always allow adequate space for individual, anonymous judgments (Sutherland and Burgman 2015). Note that the ultimate goal should be to clarify the causes of disagreements, not necessarily to achieve consensus, particularly where fundamental value differences exist.


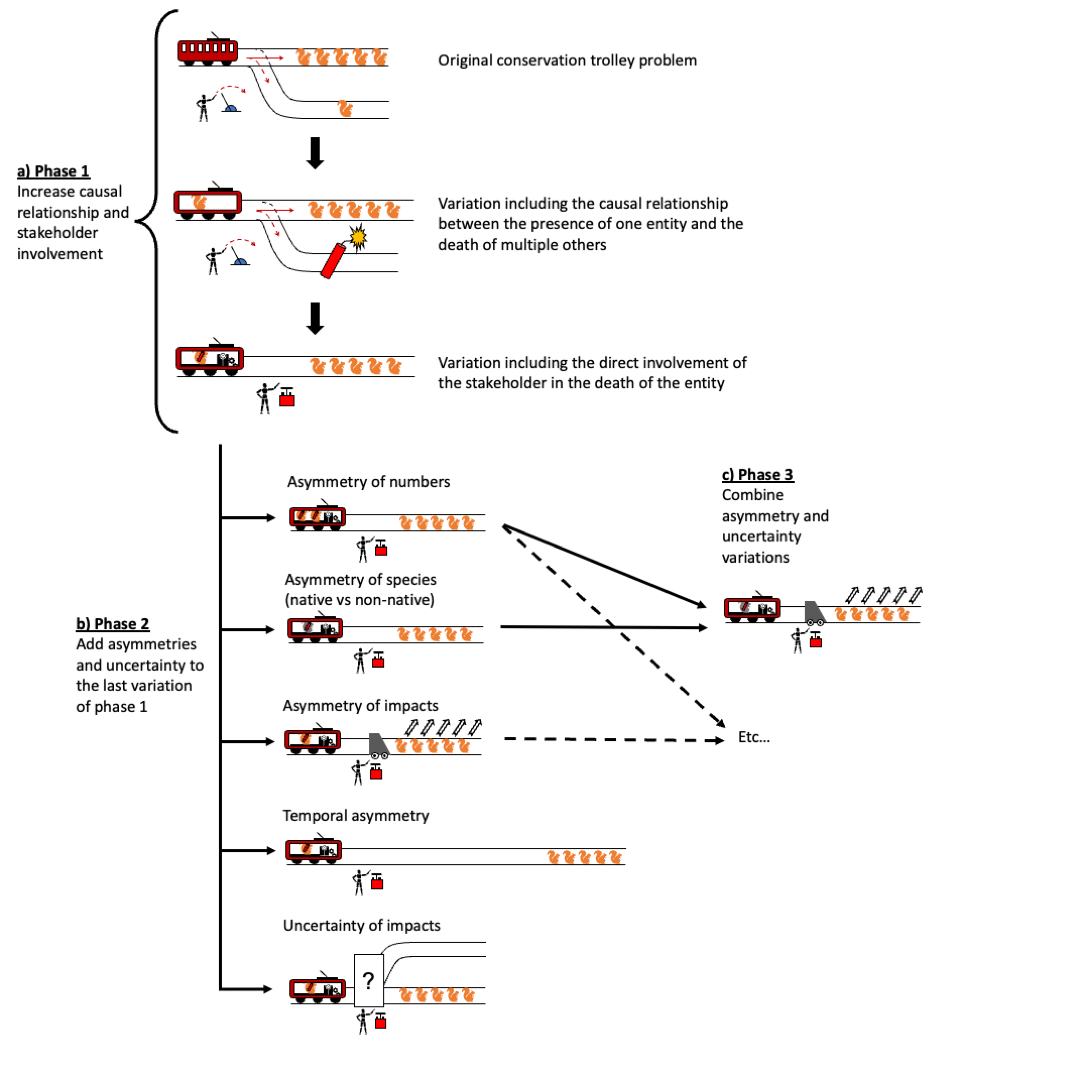


**Figure S1.** Three-phase framework for combining conservation trolley problem variations and detecting the source of conflicts of opinion or dilemma about conservation actions, using an invasion by the grey squirrel (*Sciurus carolinensis*), native to North America and introduced in various locations in Europe during the late nineteenth and the twentieth century, now threatening the native European red squirrel (*Sciurus vulgaris*) (Bertolino 2008). a) Phase 1 starts with the original conservation trolley problem, and variations are then designed to consider the relationship between the presence of an individual and the death of other squirrels (here using the variations presented in the main text for invasive alien species). Note that at this stage, we do not differentiate between species, as the goal is to examine if causal relationships are at the origin of the conflicts, while excluding potential differences in value. In the figure, subsequent variations incorporate the fact that one individual is responsible for the death of others, and that it is killed as a side effect of saving the others (e.g. through the management of the environment - first variation), and that it is killed directly (lethal control - second variation). b) In phase 2, the last variation of phase 1 is used as a basis to incorporate different elements of asymmetry and uncertainty. c) In phase 3, the variations from phase 2 are combined iteratively.

Appendix S2. Stakeholders as part of the system

In the variations and examples above, we only compared choices where stakes were directed at non-human entities of nature. In practice, conservation decisions will often also affect human beings. For example, the introduction of non-native species is often linked to economic interests (directly when benefitting from the introduced species themselves or indirectly from general trade when non-native species are transported as stowaways or contaminants). Thus, preventing the introduction of non-native species may have economic impacts such as foregone revenue from harvesting the non-native species or from causing additional economic costs for biosecurity or reduced trade volume.

The inclusion of humans into these dilemmas (Figure S2) will likely affect decisions. The difference in intrinsic value that is attributed to humans vs non-humans is likely to be incommensurable for many people. Reciprocally, monetary benefits that may be drawn from some environmental change may be considered irrelevant compared to animal life. Daw et al. (2015), Tetlock et al. (2000) and Schwartz (2021) distinguish between ‘sacred ‘and ‘secular’ values in conservation. For sacred assigned values, such as human life, compensation for their loss is impossible, while loss of secular assigned values could be compensated. Pitting entities with secular values against each other is a common issue in everyday life (‘regular choice’ hereafter). Cost-benefit analyses are such regular choices that can be applied to conservation, in which case they require the entities of nature affected and included in the analyses to be attributed a monetary value (Naidoo and Ricketts 2006). By contrast, pitting entities with sacred and secular values (referred to as taboo trade-offs, or taboo choices) can be perceived as undercutting self-image and social identity as a moral being, and generate negative cognitive, emotional, and behavioral reactions from actors facing such decisions (Fiske and Tetlock 1997). By contrast, “tragic choices” pitting entities with sacred values against each other and for which no option is satisfying, are deemed more acceptable than taboo choices, and even virtuous by people (Schoemaker and Tetlock 2012). As the type of value (sacred or secular) attributed to different entities of nature can vary between people, different stakeholders may face regular, tragic or taboo choices, potentially leading to conflict.

When secular goals have a wider or long-term effect on entities with sacred values, taboo choices may be reframed as tragic choices, potentially facilitating discussion and exchange (Daw et al. 2015). For example, impacts on non-human species and the environment can be linked to current human livelihood and that of future generations. Finally, the fact that choices may impact decision makers themselves or not (Figure S2a,b) may add another level of complexity to the dilemma.

Environmental deterioration is often linked to economic benefits (e.g. mining activities typically have negative environmental impacts but generate jobs, resources, and profits). We have focused on choices between negative outcomes, which is naturally captured by the original trolley problem. Benefits (including offsets) could be easily incorporated in the conservation trolley problem by suggesting to receive money or another reward for rerouting the trolley towards an entity (Figure S2c). Assuming the reward has a secular value, the nature of the dilemma will then depend on if the non-human entities of nature that are being impacted have a secular value (regular choice) or a sacred one (taboo choice) for the decision-makers and stakeholders.

**Figure S2.** In some situations, management actions will affect stakeholders, negatively or positively. Negative impacts will usually not be lethal, and with some uncertainty, but may affect welfare, for example, when access to some areas is restricted. a) Decision makers can be directly affected by their decision. b) The decision may affect other stakeholders than the decision makers. c) There can be positive consequences, such as profits or other rewards, associated with negative impacts on non-human entities. Depending on how values attributed to non-human entities, stakeholders, and rewards may differ from one another (have a secular or sacred value), this can lead to regular, taboo or tragic choices.

References

Bertolino S. 2008. Introduction of the American grey squirrel (Sciurus carolinensis) in Europe: a case study in biological invasion. Current Science 95: 903–906.

Canessa S, Converse SJ, West M, Clemann N, Gillespie G, McFadden M, Silla AJ, Parris KM, McCarthy MA. 2016. Planning for ex situ conservation in the face of uncertainty. Conservation Biology 30: 599–609.

Daw TM, Coulthard S, Cheung WWL, Brown K, Abunge C, Galafassi D, Peterson GD, McClanahan TR, Omukoto JO, Munyi L. 2015. Evaluating taboo trade-offs in ecosystems services and human well-being. Proceedings of the National Academy of Sciences 112: 6949–6954.

Fiske AP, Tetlock PE. 1997. Taboo Trade-offs: Reactions to Transactions That Transgress the Spheres of Justice. Political Psychology 18: 255–297.

Naidoo R, Ricketts TH. 2006. Mapping the economic costs and benefits of conservation. PLoS biology 4: e360.

Pluess T, Cannon R, Jarošík V, Pergl J, Pyšek P, Bacher S. 2012. When are eradication campaigns successful? A test of common assumptions. Biological Invasions 14: 1365–1378.

Schoemaker PJH, Tetlock PE. 2012. Taboo Scenarios: How to Think about the Unthinkable. California Management Review 54: 5–24.

Schwartz MW. 2021. Conservation lessons from taboos and trolley problems. Conservation Biology 35: 794–803.

Schwartz MW, Cook CN, Pressey RL, Pullin AS, Runge MC, Salafsky N, Sutherland WJ, Williamson MA. 2018. Decision Support Frameworks and Tools for Conservation. Conservation Letters 11: e12385.

Sutherland WJ, Burgman M. 2015. Policy advice: Use experts wisely. Nature 526: 317–318.

Tetlock PE, Kristel OV, Elson SB, Green MC, Lerner JS. 2000. The psychology of the unthinkable: Taboo trade-offs, forbidden base rates, and heretical counterfactuals. Journal of Personality and Social Psychology 78: 853–870.
